# Classifying the structural heterogeneity of peritoneal adhesions by histopathological extracellular matrix characteristics

**DOI:** 10.1007/s00423-026-04130-2

**Published:** 2026-07-10

**Authors:** Marcella Steffani, Jara Tigges, Zoé Clees, Katharina Garhammer, Philipp-Alexander Neumann, Dirk Wilhelm, Marc Martignoni, Norbert Hüser, Rebekka Dimpel, Daniel Reim, Marcus Feith, Bianca Grosser, Helmut Friess, Marie-Christin Weber

**Affiliations:** 1 Department of Surgery, TUM School of Medicine and Health, TUM University Hospital ,Technical University of Munich, Munich, Germany; 2 Institute for Advanced Study, Technical University of Munich, Munich, Germany; 3 Institute of Pathology, TUM School of Medicine and Health, Technical University of Munich, Munich, Germany; 4 Pathology, Faculty of Medicine, University of Augsburg, Augsburg, Germany; 5 Department of Surgery, TUM School of Medicine and Health, TUM University Hospital, Technical University of Munich, Munich, Germany

**Keywords:** Peritoneal adhesions, Adhesions, General surgery, Bowel obstruction

## Abstract

**Background:**

Peritoneal adhesions are a common complication following abdominal surgery and contribute substantially to long-termmorbidity, including bowel obstruction, chronic pain, and infertility. Their diverse clinical presentation, ranging from thin strands todense obstructive bands, suggests multiple underlying pathomechanisms. We propose a histopathological framework for classifyingperitoneal adhesions based on extracellular matrix characteristics, providing a microscopic correlation to clinical heterogeneity andsupporting translational research on adhesion prevention.

**Methods:**

In this study, adhesions from 77 patients were examined histologically. Masson's Trichrome staining was used to visualize the extracellular matrix, enabling classification into five clusters based on qualitative histological analysis: stringy, dense, loose, fatty, and mixed.

**Results:**

The "stringy" and "dense" clusters exhibited the highest extracellular matrix fraction and vessel area, whereas the"loose" and "fatty" clusters displayed lower ECM density and vascularity. Immunohistochemical staining for αSMA (myofibroblasts) and PGP9.5 (nerve fibers) further revealed cluster-specific differences in myofibroblast presence and innervation. Cluster distribution was significantly associated with clinical parameters, including adhesion load and prior peritonitis.

**Conclusions:**

This is the first study to systematically categorize peritoneal adhesions byextracellular matrix characteristics, identifying five distinct histopathological phenotypes. Our findings highlight the structural andcellular diversity of adhesions, offering new insights with potential implications for understanding adhesion pathophysiology andguiding future therapeutic strategies. Validation in larger cohorts will be essential to confirm this framework and establish its clinicalutility.

**Supplementary Information:**

The online version contains supplementary material available at 10.1007/s00423-026-04130-2.

## Introduction

Abdominal peritoneal adhesions are a recognized cause of long-term complications following abdominal surgery, intra-abdominal infections, and other inflammatory conditions, including small bowel obstruction, chronic abdominal pain, and secondary infertility [1, 2]. Despite recent advances in understanding the cellular pathophysiology of peritoneal adhesion formation, effective therapeutic measures to completely prevent peritoneal adhesions and the related complications have not been widely established in surgical practice yet [3].

Over 90% of patients undergoing intra-abdominal surgery develop peritoneal adhesions, which can lead to the adhesion-related morbidities mentioned above [4, 5] . A high rate of patients is readmitted to the hospital within the first year after intra-abdominal surgery for adhesion-related complications [1, 2, 6]. Even in cases of asymptomatic peritoneal adhesions, a high adhesion load can lead to a significantly higher rate of intra- and postoperative complications in patients undergoing abdominal reoperation [7].

Peritoneal adhesions can be defined as extensive scar tissue that forms between organs within the peritoneal cavity following intra-abdominal surgery or infection. Known pathophysiological processes involved in peritoneal adhesion formation include lack of fibrinolytic activity, excessive aggregation of peritoneal macrophages and mesothelial activation, ultimately leading to fibrotic conversion during the post-operative healing process [8]. Insights into serosal healing processes and the role of peritoneal macrophages and mesothelial cells in the development of peritoneal adhesions have revolutionized the understanding of the pathophysiology of peritoneal adhesions in recent years [9–13].

However, it is well known from surgical practice that peritoneal adhesions can exhibit a remarkable variability in form and severity. The spectrum of peritoneal adhesions routinely encountered in clinical practice ranges from isolated, cord-like bands of fibrous tissue, to diffuse, easily separable adhesions, to extensive, dense adhesions involving multiple organs. Clinical knowledge about the significant heterogeneity of peritoneal adhesions regarding their form and extent highlights the complexity of the underlying pathophysiological mechanisms, yet they remain incompletely understood.

Currently, clinical scoring systems for peritoneal adhesions include the Peritoneal Adhesion Index proposed by Coccolini et al. [14]. The score is based on macroscopic assessment of peritoneal adhesions intraoperatively according to their extent and surgical separability.

Basic and translational research into the prevention of peritoneal adhesions is often based on microscopic assessment of adhesions in preclinical animal models. To bridge the gap between clinical knowledge of the macroscopic heterogeneity of peritoneal adhesions and histological evaluation of peritoneal adhesions in basic research, the aim of this study was to develop a histopathological framework to distinguish microscopically the different clinical manifestations of peritoneal adhesions.

The main focus on distinguishing subtypes of adhesions is the assessment of extracellular matrix (ECM) characteristics, as scarring and ECM deposition represent the final phenotype of peritoneal adhesions. Based on the identified histological phenotypes, five clusters of adhesions were identified. These clusters could serve as a basis for basic research to link specific pathomechanisms to the clinical manifestations of adhesions, thereby expanding our understanding of the pathophysiology behind the variability of adhesions in form and severity.

## Methods

### Patient samples

Histological sections of peritoneal adhesions from 77 patients undergoing elective or emergency abdominal surgery at the Department of Surgery, TUM University Hospital, TUM Klinikum Rechts der Isar, Technical University of Munich, were included in the study. The study was performed according to the Declaration of Helsinki. The study protocol was approved by the Ethics Committee of the Technical University of Munich, TUM School of Medicine and Health (95/22 S-KK). Informed consent was obtained from patients to participate in the study. No prospective intraoperative selection of specific adhesion phenotypes was performed.

### Histology

Adhesiolysis is typically performed either during emergency surgery for mechanical bowel obstruction or during elective procedures to obtain safe access to the operative field. When adhesion bands are encountered, they are routinely resected en bloc to remove the scar tissue from the abdominal cavity, in accordance with the standard protocol in our clinic. The resected specimens were immediately placed in 4% paraformaldehyde and transferred to the laboratory within 20 min to minimize post-excision artefacts. All samples were uniformly processed, embedded, and sectioned by the same investigator to avoid variability introduced by fixation, embedding, or sectioning procedures. Sections of formalin-fixed, paraffin-embedded (FFPE) histologic samples from human peritoneal adhesions were used for histologic staining as described below with section thickness of 2.5 μm. Histological sections were stained with Masson’s trichrome with Aniline Blue (Morphisto GmbH, Germany) according to the manufacturer’s instructions. In patients from whom more than one adhesion specimen was collected intraoperatively due to a high adhesion load, all specimens were embedded together in the same histological block. Samples in which all individual adhesions exhibited the same characteristics were assigned to the corresponding cluster, whereas samples showing heterogeneity among individual adhesions within a single block were classified into the mixed cluster.

Stained slides were scanned at 400x magnification using a digital pathology scanner (Aperio AT2, Leica Biosystems). The pathology slide viewing software Aperio ImageScope (Leica Biosystems) and QuPath were used to visualize scanned histologic images.

### Immunohistochemistry

Heat-mediated antigen-retrieval was performed after deparaffinization using citrate buffer at pH 6 for 10 min for immunohistochemistry. For tissue permeabilization, histological sections were incubated with TritonX100 0.5% for 7 min, and endogenous peroxidase activity was deactivated with 3% hydrogen peroxide in methanol for 5 min. Histological sections were blocked with 2% bovine serum albumin for 1 h. Primary antibodies anti-alpha smooth muscle actin (αSMA) mouse polyclonal antibody (Abcam, ab7817, 1:500) or anti-Protein Gene Product 9.5 (PGP9.5) rabbit polyclonal antibody 1:500 (Invitrogen, #PA5-29012, 1:500) were incubated overnight at 4 °C. HRP-linked secondary antibodies (Jackson ImmunoResearch, 111-035-003 or 115-035-003, 1:1000) were incubated for 1 h at room temperature prior to DAB reaction. Slides were counterstained with Mayer’s hematoxylin. One negative control without the primary antibody was included for each adhesion sample to rule out nonspecific staining. As a positive control for the staining, transmural histological sections of small intestine were used.

### Histopathological classification of peritoneal adhesions

Masson’s trichrome-stained histological sections were first qualitatively evaluated with respect to the characteristics of the extracellular matrix (ECM). Both the general shape of the adhesions and the overall ECM density were assessed. Based on these features, the adhesions were assigned to four primary clusters. The first cluster, “stringy,” comprised adhesions with a string-like phenotype, representing band-like adhesions with predominantly dense ECM. Broader, more extensive adhesions were classified either as “dense,” representing broad adhesions with tightly packed ECM, or as “loose,” representing broad adhesions with loosely packed ECM. Adhesions consisting mainly of adipose tissue were classified as “fatty.” A fifth cluster “mixed” was introduced for adhesions with an inconsistent phenotype that displayed more than one of the characteristics described above, or for cases in which assignment to a single cluster could not be reliably determined.

### Histomorphometry

**Fig. 1:**
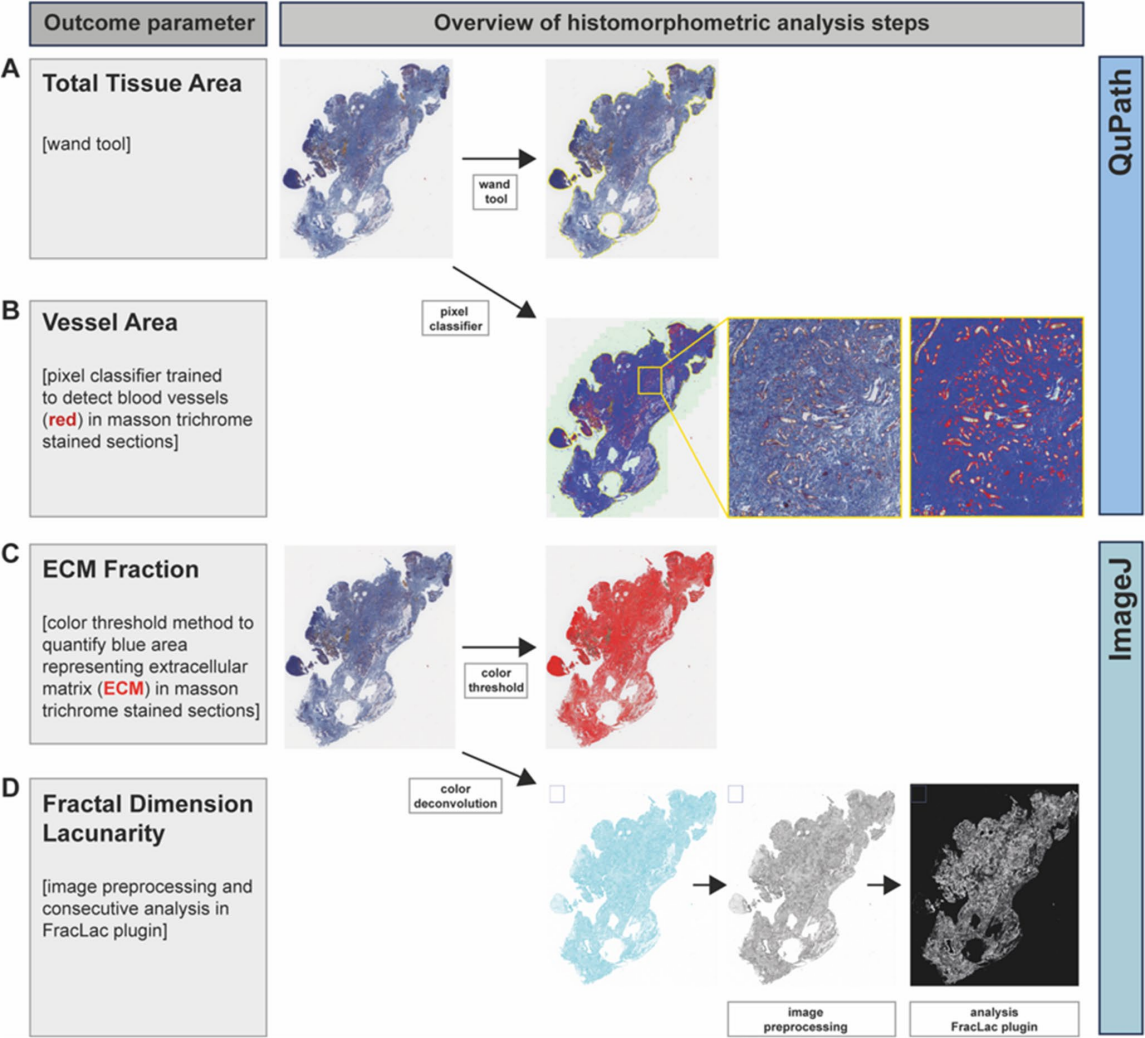
Overview of histomorphometric analysis steps.(**A**) The total tissue area was measured using the wand tool in QuPath. (**B**) The vessel area was measured using the pixel classfier in QuPath to detect blood vessels within the histological section, priorly trained by the investigators. (**C**) The extracellular matrix (ECM) fraction was measured using the color threshold method in FIJI/ImageJ to detect only the blue areas in the Masson’s trichrome stained sections indicating ECM. (**D**) The fractal dimension and lacunarity as measures for the structural complexity of the ECM were determined using the FracLac plugin in FIJI/ImageJ after image preprocessing

Histomorphometrical analysis of the Masson’s trichrome stained histological sections was performed using QuPath [15] and FIJI/ImageJ [16]. An overview of the histomorphometric analysis steps and outcome parameter is given in Fig. 1. In a first step, the total tissue area was assessed as a reference for consecutive histomorphometric parameters including the vessel area and extracellular matrix (ECM) fraction. To measure the total tissue area, the wand tool in QuPath was applied to outline the tissue within the histological section (Fig. 1A). In the next step, a pixel classifier was trained in QuPath to measure vessel area by detecting blood vessels while excluding stroma, fat cells, nuclei, erythrocytes, and the vessel lumen. The pixel classifier in QuPath is a machine-learning–based tool that classifies individual pixels in a histological image according to their visual features and can be used to segment tissues. The pixel classifier was trained by the investigators based on 10 exemplary histological adhesion scans. The vessel area was then defined as the percentage of the vessel annotation area relative to the total tissue area (Fig. 1B). The histological image was then exported to ImageJ to measure the ECM fraction using the color threshold method. To do so, a color threshold was applied to select the ECM area (blue) in the Masson’s trichrome stained histological sections. The ECM fraction was defined as the percentage of the ECM area to the total tissue area (Fig. 1C). Fractal dimension and lacunary as parameters for the structural complexity of the ECM were assessed using the FracLac Plugin in ImageJ [17, 18] after image preprocessing as previously described (Fig. 1D) [19, 20].

### Assessment of immunohistochemistry stainings

All 77 histological samples were stained for αSMA as a myofibroblast marker and PGP9.5 as a neuronal marker. Histological samples were either classified as positive or negative for αSMA+ myofibroblasts or PGP9.5 + cells by visual assessment of the immunohistochemically stained samples. The entire histological sections was assessed using visualization of the stained slides and negative controls (no primary antibody) in QuPath. In detail, the presence of myofibroblasts within the adhesions was assumed when individual spindle-shaped, DAB-positive cells were observed in the ECM of samples immunostained for αSMA. If αSMA immunostaining showed only αSMA-positive muscular cell bundles or smooth muscle cells and pericytes surrounding blood vessels, these samples were classified as negative for αSMA+ myofibroblasts. If cells positive for PGP9.5 were present within the adhesion samples, these samples were classified as PGP9.5+.

### Clinical patient data

In addition to patients’ histology, we collected retrospective clinical data. Demographic and clinicopathologic characteristics of patients were obtained from medical records. Variables collected included sex, age, medical history, previous surgeries, time from previous surgeries, previous complications including history of peritonitis, type of surgery, and underlying condition.

### Statistical analysis and data visualization

GraphPad Prism (version 10.1.0; GraphPad Software) and SPSS (version 20.0.9.2; IBM) were used for data visualization and statistical analysis. Statistical differences were calculated using the one-way ANOVA with Tukey’s multiple comparison test for normally distributed histomorphometric data. Fisher’s exact test and Pearson‘s Chi-square test were used for contingency analysis. *p*-value of < 0.05 was considered statistically significant.

## Results

### Patient characteristics

Histological sections from peritoneal adhesions from 77 patients (43 male; 34 female) were included in the study. The indication for surgery was small bowel obstruction in 19.5%, enteric fistula or anastomotic leakage in 7.8%, elective surgery for benign diagnoses (enterostomy reversal, herniotomy) in 42.9%, elective surgery for malign diagnosis (cancer resections) in 18.1% and others in 11.7% of cases (Table 1). 79.2% of surgeries were elective surgeries. The mean number of previous abdominal surgeries was 3, with a range from 0 to 12. The mean time from previous surgery was 45 (range 1–600) months. 54.5% of patients had a history of peritonitis. The adhesion load was categorized into single adhesions and multiple adhesions with 81.8% of patients presenting with multiple adhesions (Table 1).

**Table 1:** Patient characteristics of study cohort

Gender	Frequency in % (*n*) or mean [21] *n* = 77
- male	55.8 (43)
- female	44.2 (34)
Age	
	62 (23–81)
Indication for surgery	
- bowel obstruction	19.5 (15
- enteric fistula/anastomotic leakage	7.8 (6)
- elective surgery (benign diagnosis): enterostomy reversal, herniotomy	42.9 (33)
- elective surgery (malign diagnosis): cancer resection	18.1 (14)
- other	11.7 (9)
Urgency of surgery	
- elective	79.2 (61)
- emergency	20.8 (16)
Number of previous surgeries [n]	3 (0–12)
Time from previous surgery [months]	45 (1–600)
History of peritonitis	
- no	54.5 (42)
- yes	45.5 (35)
Adhesion load	
- single	18.2 (14)
- multiple	81.8 (63)
Histological cluster	
- stringy	18.2 (14)
- dense	27.2 (21)
- loose	18.2 (14)
- fatty	10.4 (8)
- mixed	26 (20)

### Histopathological classification of peritoneal adhesions

**Fig. 2:**
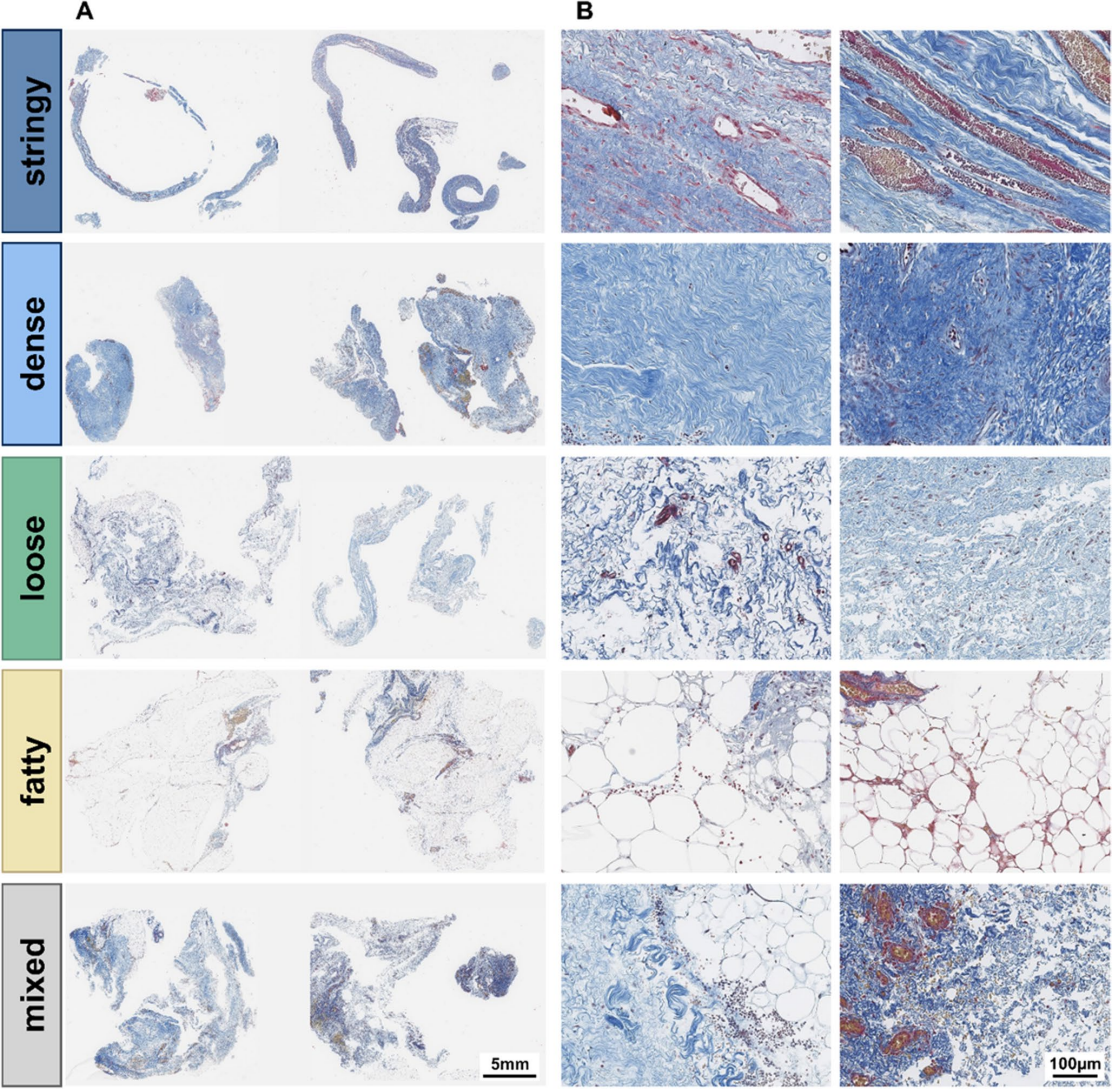
Adhesion clusters.(**A**) Overview images of Masson’s trichrome stained sections of human adhesions with two representative sections per cluster (“stringy”, “dense”, “loose”, “fatty” and “mixed”). Extracellular matrix (ECM) is stained blue by Masson’s trichrome stain. Scale bar = 5 mm. (**B**) Detailed sections from the histological overview images illustrating the morphology of the structure of the extracellular matrix. Scale bar = 100 μm

In a first step, histological samples were qualitatively assessed with regard to ECM characteristics as described in detail in the methods section. Summarized, the following features were evaluated: the general shape of the adhesion and the ECM density. Based on these characteristics, we defined five clusters (Fig. 2; Supplementary Figs. 1–5). Each adhesion sample was assigned to one of these clusters during the qualitative histological assessment.

- Cluster 1 (“Stringy”): string-like adhesions with mainly dense ECM.
- Cluster 2 (“Dense”): broad adhesions with densely packed ECM.
- Cluster 3 (“Loose”): broad adhesions with loosely organized ECM.
- Cluster 4 (“Fatty”): adhesions mainly consisting of fat tissue.
- Cluster 5 (“Mixed”): adhesions with mixed characteristics.

Of the 77 samples 14 were characterized as “stringy”, 21 as “dense”, 14 as “loose”, 8 as “fatty” and 20 as “mixed” (see Supplementary Figs. 1–5 for histological images of all adhesion samples).

To assess the reproducibility of the cluster assignment, histological sections were independently classified by a second and third investigator blinded to all clinical data. The “mixed” cluster, which comprised of samples displaying features of more than one primary phenotype, should be interpreted as representing a heterogeneous category.

### Histomorphometric characterisation of adhesion clusters

In a second step, histomorphometric characterization of the histological sections was conducted to objectively quantify structural features of the ECM within the qualitatively defined clusters. Vessel area was assessed as a measure of tissue vascularization, fractal dimension and lacunarity as measures of the structural complexity and spatial heterogeneity of the ECM, and ECM fraction as a measure of overall ECM density.

**Fig. 3:**
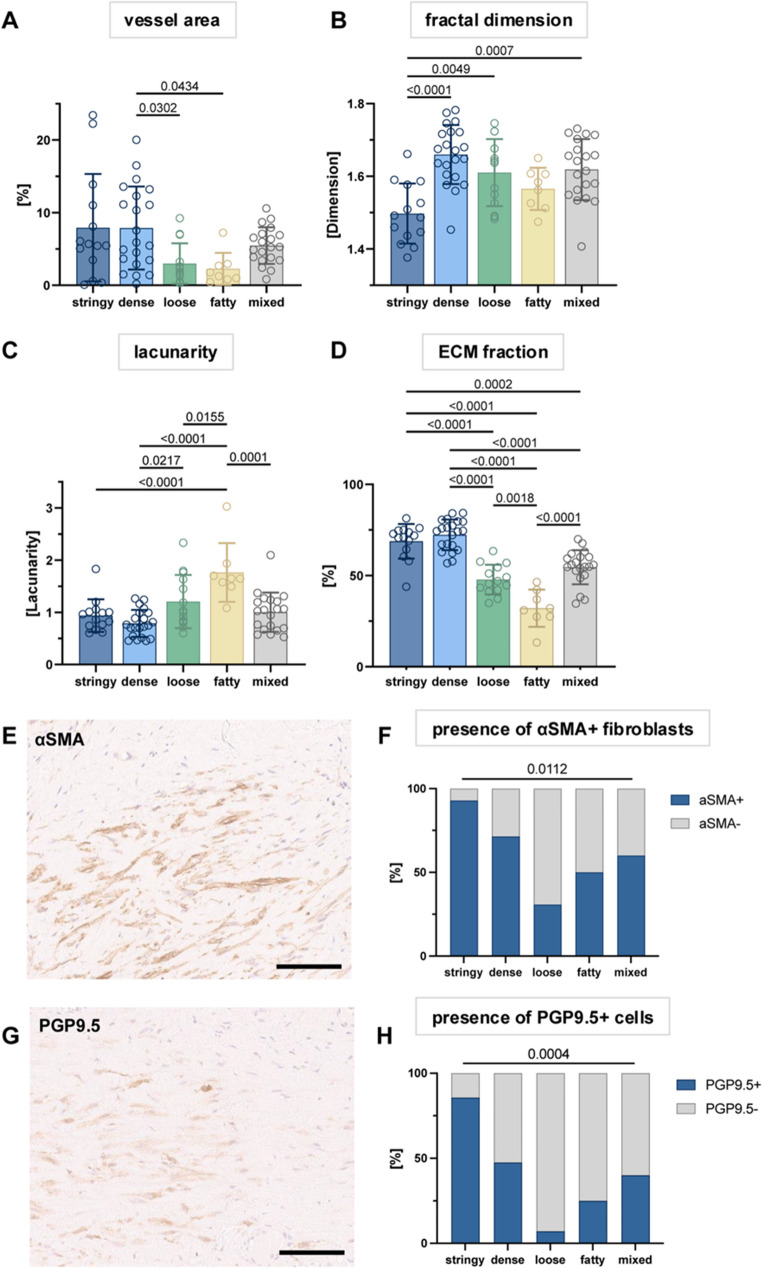
Histomorphometric analysis and immunohistochemistry of peritoneal adhesions.(**A**) Vessel area in %, (**B**) fractal dimension, (**C**) lacunarity, and (**D) **extracellular matrix (ECM) fraction in % are presented for each cluster. Ordinary one-way ANOVA with multiple comparisons and Tukey correction. (**E**) Examplary image of immunohistochemistry staining for alpha smooth muscle actin (αSMA) showing αSMA+ myofibroblasts in adhesion sample. Scale bar = 100 μm, 400x magnification. (**F**) Presence of αSMA+ myofibroblasts in adhesion sample per cluster. Fisher‘s exact test. (**G**) Examplary image of immunohistochemistry staining for the neuronal marker Protein Gene Product 9.5 (PGP9.5) in adhesion sample. (**H**) Presence of PGP9.5 + cells in adhesion samples per cluster. Fisher‘s exact test

The mean vessel area was 7.93% in the “stringy”, 7.89% in the “dense”, 3.00% in the “loose”, 2,27% in the “fatty” and 5.46% in the “mixed” cluster (Fig. 3A).

Regarding the density of the ECM, the ECM fraction was lowest in the “loose” cluster (47.79%) and “fatty” cluster (32.10%) and highest in the “dense” cluster (72.32%) (Fig. 3D).

Quantification of the structural complexity of the ECM by fractal analysis revealed the “dense” cluster to have the highest structural complexity with a mean fractal dimension of 1.66 and the “stringy” cluster to have the lowest fractal dimension of 1.50 (Fig. 3B).

The lacunarity as a measure for the porosity of the ECM was lowest in the “dense” cluster with a mean of 0.94 and highest in the “fatty” cluster with a mean of 1.76, followed by the “loose” cluster with a mean of 1.21 (Fig. 3C).

### Identification of myofibroblasts and nerve tissue in adhesions

Immunohistochemical staining for αSMA was performed to identify myofibroblasts within adhesions. Adhesions were characterized as either positive or negative for the presence of myofibroblasts depending on the presence of αSMA+ cells within the stroma (Fig. 3E). The distribution of αSMA-positive and -negative adhesions differed significantly between clusters (*p* = 0.0112, Fig. 3F). 92.9% of adhesions of the “stringy” cluster, 71.4% of adhesions in the “dense” cluster, and only 30.8% of adhesions in the “loose” cluster contained αSMA+ myofibroblasts.

Immunohistochemical staining for the neuronal marker PGP9.5 was performed to characterize whether there was nerve tissue within the adhesion (Fig. 3G). Again, the distribution of adhesions positive or negative for PGP9.5 was significantly different between clusters (*p* = 0.0004, Fig. 3H). 85.7% of adhesions in the “stringy”, 47.6% in the “dense”, 7.1% in the “loose”, 25.0% in the “fatty”, and 40.0% of adhesions in the “mixed” cluster were positive for PGP9.5 (Fig. 3H).

### Correlation of adhesion clusters to clinical characteristics

**Fig. 4:**
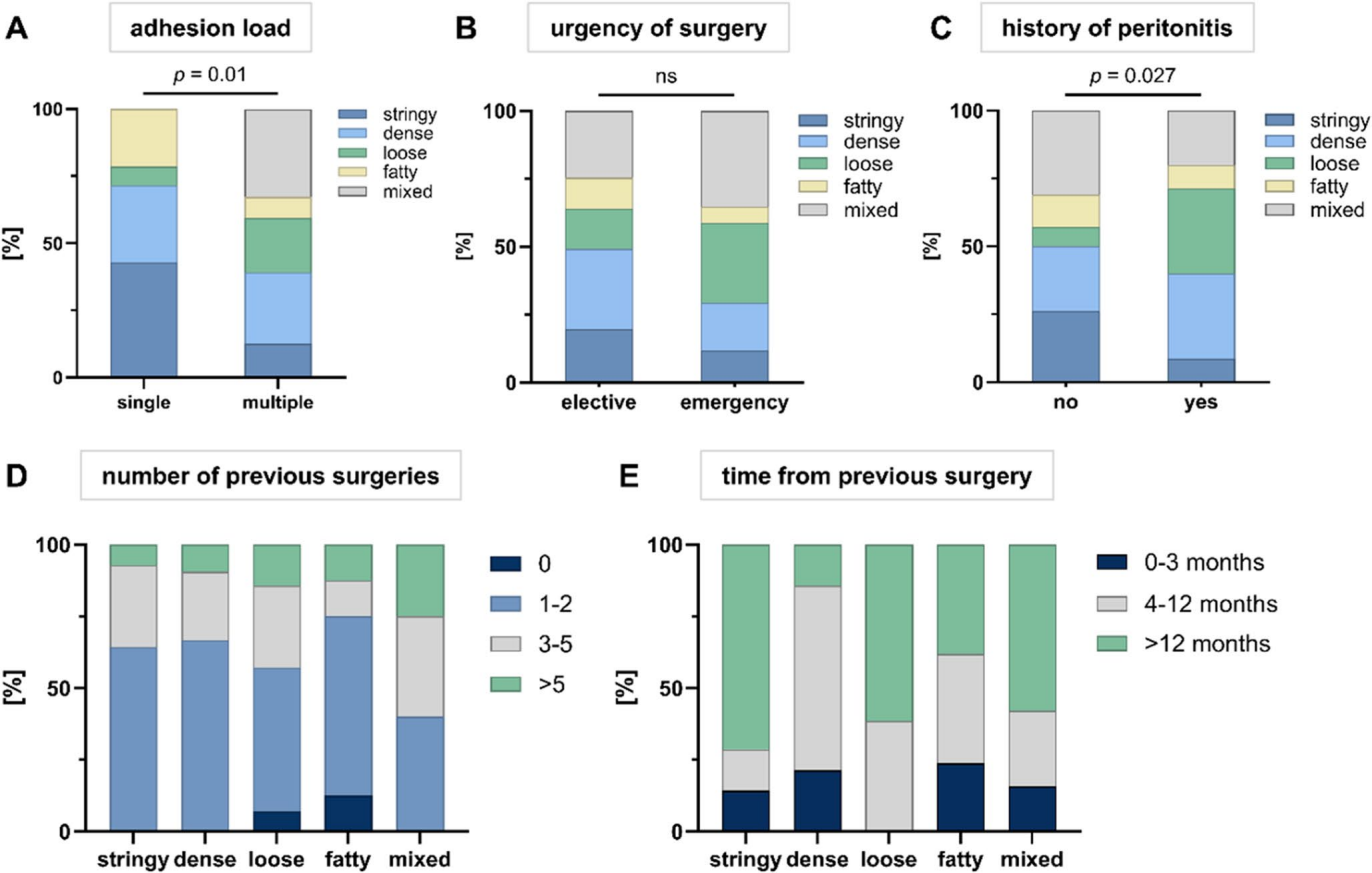
Correlation of clusters with clinical data. (**A**) Cluster distribution according to intraoperative adhesion load (single adhesions vs. multiple adhesions). (**B**) Cluster distribution according to urgency of surgery (elective vs. emergency surgery). (**C**) Cluster distribution according to history of peritonitis. (**A-C**) Pearson‘s Chi-square test. (**D**) Number of previous surgeries per cluster.** (D**) Time from previous surgery per cluster

The distribution of adhesion clusters in relation to the adhesion load showed significant differences (Fig. 4A). When single adhesions were present, they were from the “stringy” cluster in 42.9% and from the “dense” cluster in 28.6%. The cluster “mixed” was only found in patients with multiple adhesions and thus high adhesion load (Fig. 4A).

There was no significant correlation between clusters and the indication for surgery (*p* = 0.720) and the urgency of surgery (*p* = 0.502) (Table 2; Fig. 4B).

**Table 2:** Patient characteristics stratified by cluster

	Stringy *(n = 14)*	Dense *(n = 21)*	Loose *(n = 14)*	Fatty *(n = 8)*	Mixed *(n = 20)*	*p*-value
	Frequency in % (*n*) or mean (*n*)					
**Gender**						
- male	23.3 (10)	27.9 (12)	18.6 (8)	11.6 (5)	18.6 (8)	*0.465*
- female	11.8 (4)	26.5 (9)	17.6 (6)	8.8 (3	35.3 (12)	
**Indication for surgery**						
- bowel obstruction	6.7 (1)	13.3 (2	26.7 (4)	6.7 (1)	46.7 (7)	*0.720*
- enteric fistula/anastomotic leakage	33.3 (2	33.3 (2	0 (0)	16.7 (1)	16.7 (1)	
- elective surgery (benign diagnosis): enterostomy reversal, herniotomy	18.2(6)	36.4 (12)	15.2 (5	12.1 (4)	18.2 (6)	
- elective surgery (malign diagnosis): cancer resection	21.4 (3)	28.6 (4)	14.3 (2)	7.1 (1)	28.6 (4)	
- other	22.2 (2)	11.1 (1)	33.3 (3)	11.1 (1)	22.2 (2)	
**Urgency of surgery**						
- elective	19.7 (12)	29.5 (18)	14.8 (9)	11.5 (7)	24.6 (16)	*0.502*
- emergency	12.5 (2)	18.3 (3)	31.3 (4)	6.3 (1)	31.3 (4)	
**Number of previous surgeries** [n]	2 (1–6)	2 (1–7)	3 (0–7)	3 (0–7)	4 (1–12)	*0.473*
**Time from previous surgery** [months]	45 (1–280)	16 (1–60)	62 (4–423)	45 (3–180)	67 (1–600)	*0.174*
**History of peritonitis**						
- no	26.2 (11)	23.8 (10)	7.1 (3)	11.9 (5	31 (13)	*0.027*
- yes	8.6 (3)	31.4 (11)	31.4 (11)	8.6 (3)	20 (7)	
**Adhesion load**						
- single	42.9 (6)	28.6 (4)	7.1 (1)	21.4 (3)	0 (0)	*0.010*
- multiple	12.7 (8)	27.0 (17)	20.6 (13)	7.9 (5)	31.7 (20)	

Interestingly, “loose” adhesions were most commonly found in patients with previous history of peritonitis (31.4%) and among patients with no history of peritonitis only 7.1% of adhesions were clustered as “loose” (Table 2; Fig. 4C).

Although there was no significant difference between clusters regarding the number of previous surgeries (*p* = 0.473) and the time from previous surgery (*p* = 0.174) (Table 2; Fig. 4D**/**E), the majority of “dense” adhesions was found 4–12 months after the previous surgery. In contrast, the majority of “loose” adhesions was found > 12 months after the previous surgery (Fig. 4E).

## Discussion

In this study, histologically evaluating 77 human peritoneal adhesions samples, we showed that human peritoneal adhesions can exhibit different histological phenotypes based on their shape and ECM characteristics. We identified 5 distinct adhesion phenotypes: “stringy”, “dense”, “loose”, “fatty”, and “mixed”.

The main objective was to identify microscopic correlates to the diverse clinical spectrum of adhesions encountered by abdominal surgeons, ranging from isolated single adhesion bands to broad, extensive adhesions that may be either very soft or thick and fibrotic to bridge the gap between clinical knowledge of the macroscopic heterogeneity of peritoneal adhesions and histological assessment of peritoneal adhesions in basic research.

It is commonly known from surgical experience that the extent of adhesions during revision surgery is rather unpredictable. Not only the presence or absence of adhesions, but also their phenotype varies among patients, ranging from localized to wide-spread and from fibrous cord-like structures to cobweb-like flimsy adhesions [22]. The clinical experience of distinct stages of fibrosis in peritoneal adhesions prompted us to explore whether different histological phenotypes could be identified based on the microscopic structure of the ECM. Our findings thus highlight the variability of microscopic structural characteristics of peritoneal adhesions with special emphasis on ECM composition and organization, but also regarding differences in vascular density (measured by vessel area), nerve tissue (identified by immunostaining for PGP9.5) and myofibroblastic transformation (identified by immunostaining for αSMA).

Previous studies have described peritoneal adhesions as fibrotic bands resulting from impaired wound healing, but a detailed histopathological subclassification remains unclear [23].

Our classification of adhesions into five clusters based on qualitative assessment of ECM characteristics revealed significant differences in quantification of tissue area, vascularization, ECM fraction, and neuronal structures. The stringy and dense clusters exhibited the highest ECM fraction and vascularization, which may point towards an actively remodeling tissue environment in these phenotypes. In contrast, the loose and fatty clusters displayed a lower ECM fraction, possibly indicating a less fibrotic microenvironment. The presence of myofibroblasts (αSMA-positive) and nerve fibers (PGP9.5-positive) varied significantly across clusters, with the stringy cluster showing the highest proportion of both cell types. This finding supports previous reports that myofibroblasts and neurogenic inflammation contribute to adhesion maturation and chronic pain associated with adhesions [24, 25].

The results suggest that different pathomechanisms may drive the formation of different adhesion subtypes. The increased presence of αSMA-positive myofibroblasts in the “stringy” and “dense” clusters and their absence in the “loose” cluster suggests that myofibroblastic transformation is not present in all adhesions or that it disappears during adhesion remodelling.

The central objective of this study was to bridge the gap between basic and clinical research through histologic evaluation of patient-derived adhesion samples. We included a large number of specimens from a broad range of patients and surgical indications, encompassing both elective and emergency procedures. Previous studies have described different histological phenotypes of adhesions, including varying degrees of innervation [24, 26]. Binnebösel et al. reported that adhesions classified intraoperatively as “dense” exhibited significantly increased total collagen content and higher collagen type I/III ratios compared with those intraoperatively described as “soft,” suggesting ongoing tissue remodeling that may disrupt normal wound healing and contribute to the long-term persistence of adhesions [22]. Our study supports these observations by demonstrating, for the first time, that adhesions can also be subclassified microscopically according to their extracellular matrix characteristics. Because we did not record the intraoperative macroscopic phenotype of each adhesion, only the overall extent, we cannot directly correlate our microscopic phenotypes with their macroscopic appearance. Therefore, we can only speculate that the clusters identified here align with clinically recognized adhesion types. Given the distinct phenotypes observed, we hypothesize that adhesion formation is driven by heterogeneous pathophysiological mechanisms, and that effective prevention strategies may ultimately need to be personalized [8].

After classifying the adhesions qualitatively into clusters, we performed histomorphometry to quantify the ECM density and the structural characteristics of the ECM using fractal analysis (Fig. 3). The fractal dimension is a non-Euclidean geometrical unit that serves as a metric for the structural complexity of a given pattern [27]. Fractal analysis of ECM has been used in a variety of studies to quantify the degree of organ fibrosis with a higher fractal dimension of the ECM commonly associated with advanced stages of fibrosis [28–30]. Fractal dimension provides an observer-independent quantification method for comparing treatment groups in preclinical models and could likewise be applied to preclinical adhesion models to quantitatively analyze adhesion tissue.

Our study provides a novel framework for histopathological evaluation of peritoneal adhesions primarily based on ECM characteristics, which may serve as a basis for future translational research. By linking adhesion subtypes to underlying pathophysiological mechanisms, our study may facilitate the development of targeted anti-adhesion therapies.

However, this study has limitations. While sufficient for initial clustering, the sample size may not capture the full spectrum of adhesion variability across different patient populations. Additionally, our framework for classifying peritoneal adhesions is based on histological analysis, and further studies integrating molecular and biomechanical profiling are needed to refine adhesion subtyping. An important limitation of the present study is the absence of systematic intraoperative macroscopic adhesion phenotyping. As a consequence, a direct correlation between the histological clusters identified here and established macroscopic adhesion scoring systems, such as that proposed by Coccolini et al., could not be made, and the relationship between the microscopic clusters and the macroscopic adhesion appearance observed intraoperatively remains speculative. However, the identified clusters were correlated with available clinical parameters, providing an initial indication of their potential clinical relevance. Future studies incorporating macroscopic adhesion scoring at the time of surgery will be required to establish a direct correlation between macroscopic and microscopic adhesion phenotypes.

Fibrosis is a central feature of peritoneal adhesion formation and is reflected in the present study by differences in ECM fraction, structural complexity, and the presence of αSMA-positive myofibroblasts across adhesion clusters. Beyond myofibroblast-driven ECM remodeling, oxygen sensing and HIF-related signaling have emerged as important regulators of fibrosis and wound healing, and prior experimental studies have demonstrated that modulation of HIF-related pathways substantially influences peritoneal adhesion formation and ECM remodeling [12, 31]. While the present study was designed as a descriptive histomorphological characterization rather than an investigation of the molecular pathways underlying fibrosis, correlating markers of HIF activity, such as CA9 or HIF-1α, with the histological clusters identified here represents a priority direction for future studies. Additional aspects of the adhesion microenvironment, including macrophage infiltration, mesothelial biology, and angiogenesis, likewise warrant further molecular characterization in this context.

An important limitation of the present study is the absence of systematic intraoperative macroscopic adhesion phenotyping. As a consequence, the relationship between the microscopic clusters identified here and the macroscopic adhesion appearance observed intraoperatively remains speculative. However, the identified clusters were correlated with available clinical parameters, providing an initial indication of their potential clinical relevance. Future studies incorporating macroscopic adhesion scoring at the time of surgery will be required to establish a direct correlation between macroscopic and microscopic adhesion phenotypes.

Finally, the retrospective nature of clinical data collection may introduce bias, and prospective validation of our findings in a larger cohort is warranted, including more detailed clinical data.

In summary, this is the first study to classify peritoneal adhesions based on extracellular matrix characteristics and to quantify ECM structural complexity using fractal analysis, a technique increasingly applied to assess fibrosis in both human tissue and preclinical models [20, 30]. The findings of the study may help bridge the gap between the heterogeneity of human adhesions to preclinical models of peritoneal adhesions where scarring and extracellular matrix features can be quantified and correlated to specific pathophysiological mechanisms. Finally, this histological framework provides a foundation for future multi-omics investigations aimed at elucidating the molecular and cellular mechanisms underlying the persistence of peritoneal adhesions after abdominal surgery.

## Electronic Supplementary Material

Below is the link to the electronic supplementary material.

Supplementary Material 1 (DOCX 49.7 MB)

## Data Availability

No datasets were generated or analysed during the current study.

## Declarations

### Abstract presentations

Deutscher Chirurgie Kongress 2025, Munich, Germany.
