# Supplementary material for: Classifying the structural heterogeneity of peritoneal adhesions by histopathological extracellular matrix characteristics
Source: Langenbecks Arch Surg. 2026 Jul 10;411(1):191. doi: 10.1007/s00423-026-04130-2 (PMC13354687; doi:10.1007/s00423-026-04130-2)
Supplement: Supplementary file 1 — Supplementary Material 1 (DOCX 49.7 MB) [file 423_2026_4130_MOESM1_ESM.docx]

## **Supplementary Figures**


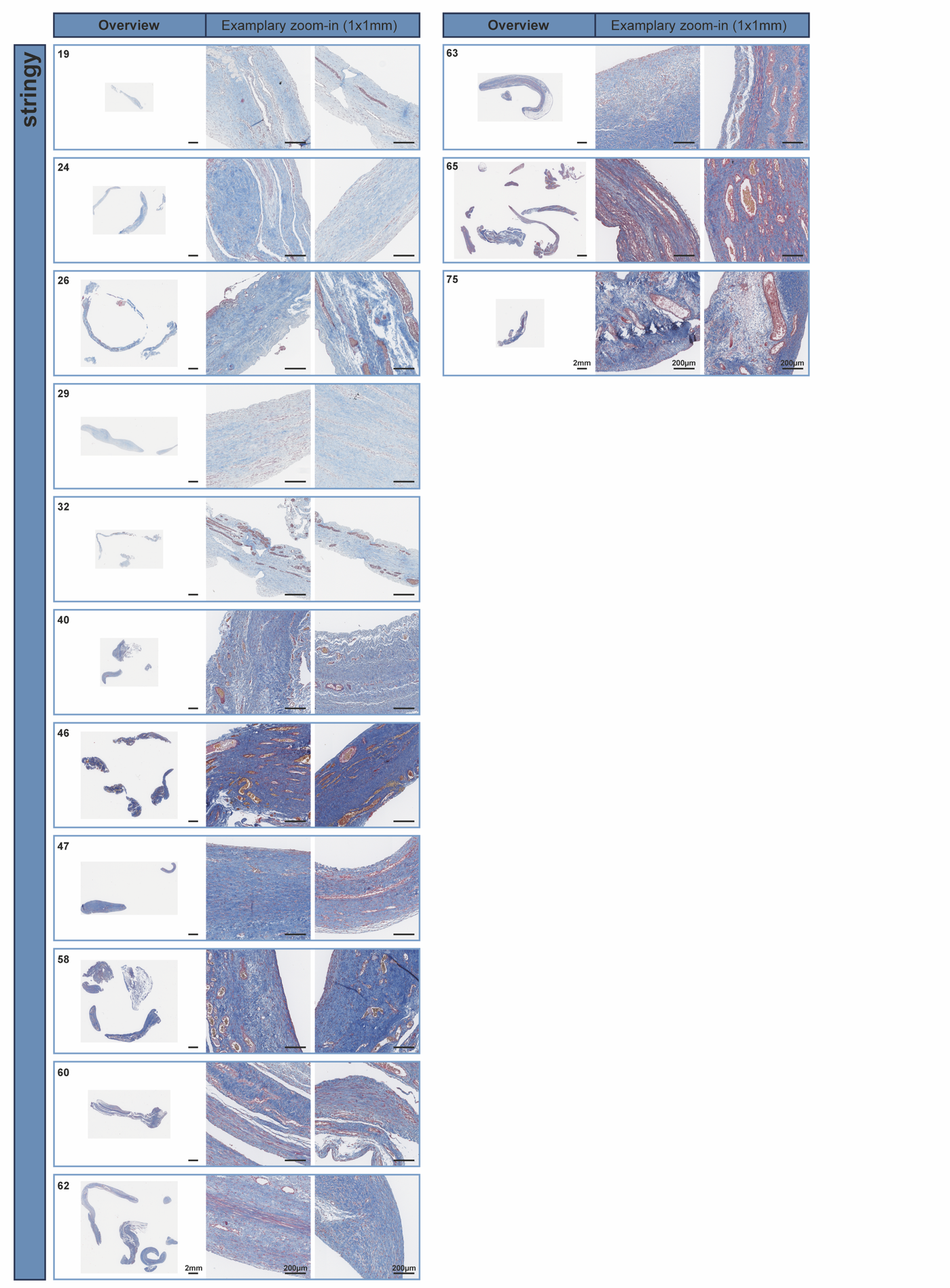


**Supplementary Figure 1:** Overview scans of all adhesion samples from the “stringy” cluster with 2 exemplary zoom-in sections (1x1mm) per sample. Scale bar = 2mm (overview) and 200µm (zoom-in).


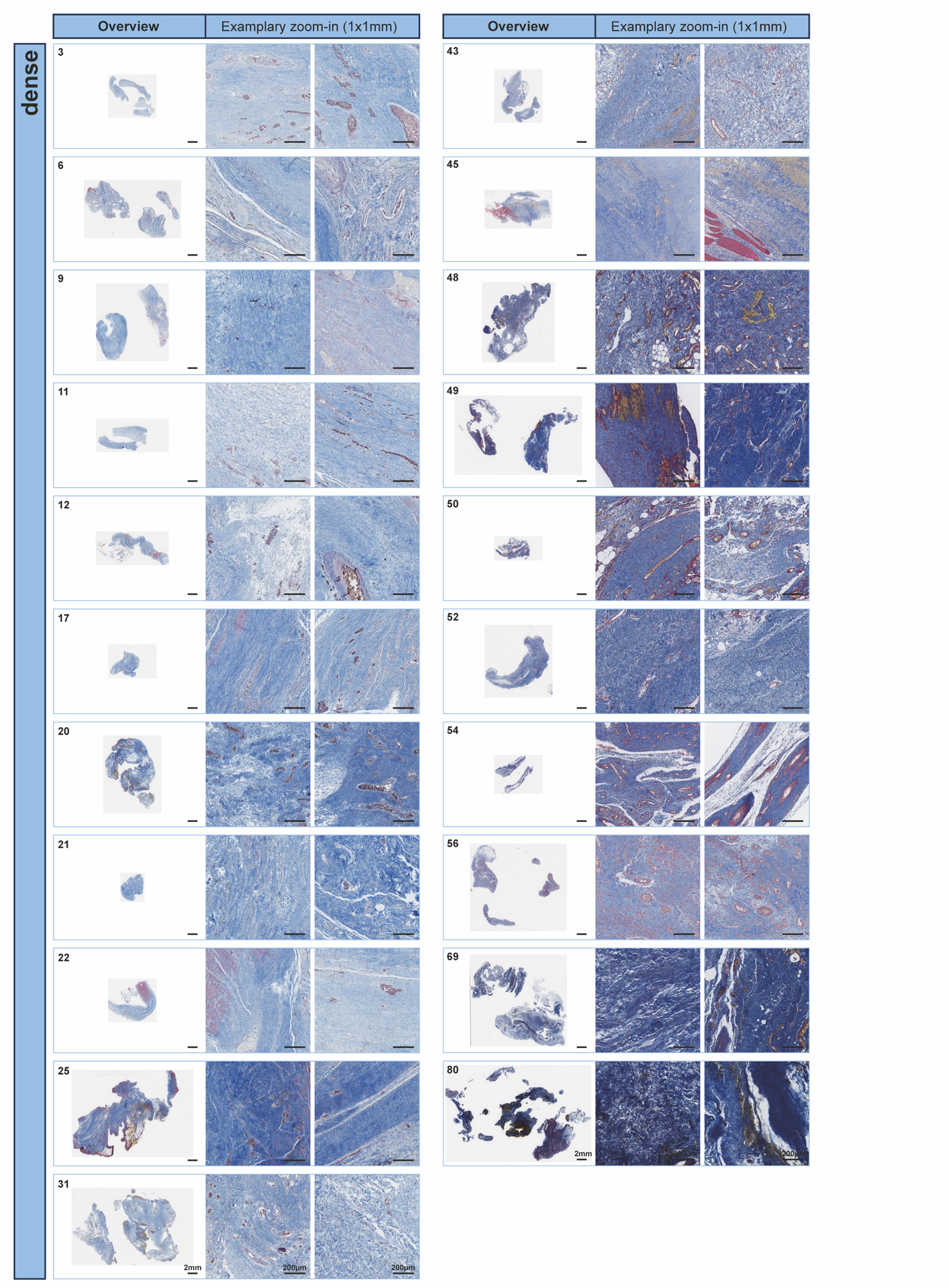


**Supplementary Figure 2:** Overview scans of all adhesion samples from the “dense” cluster with exemplary zoom-in sections (1x1mm) per sample. Scale bar = 2mm (overview) and 200µm (zoom-in).


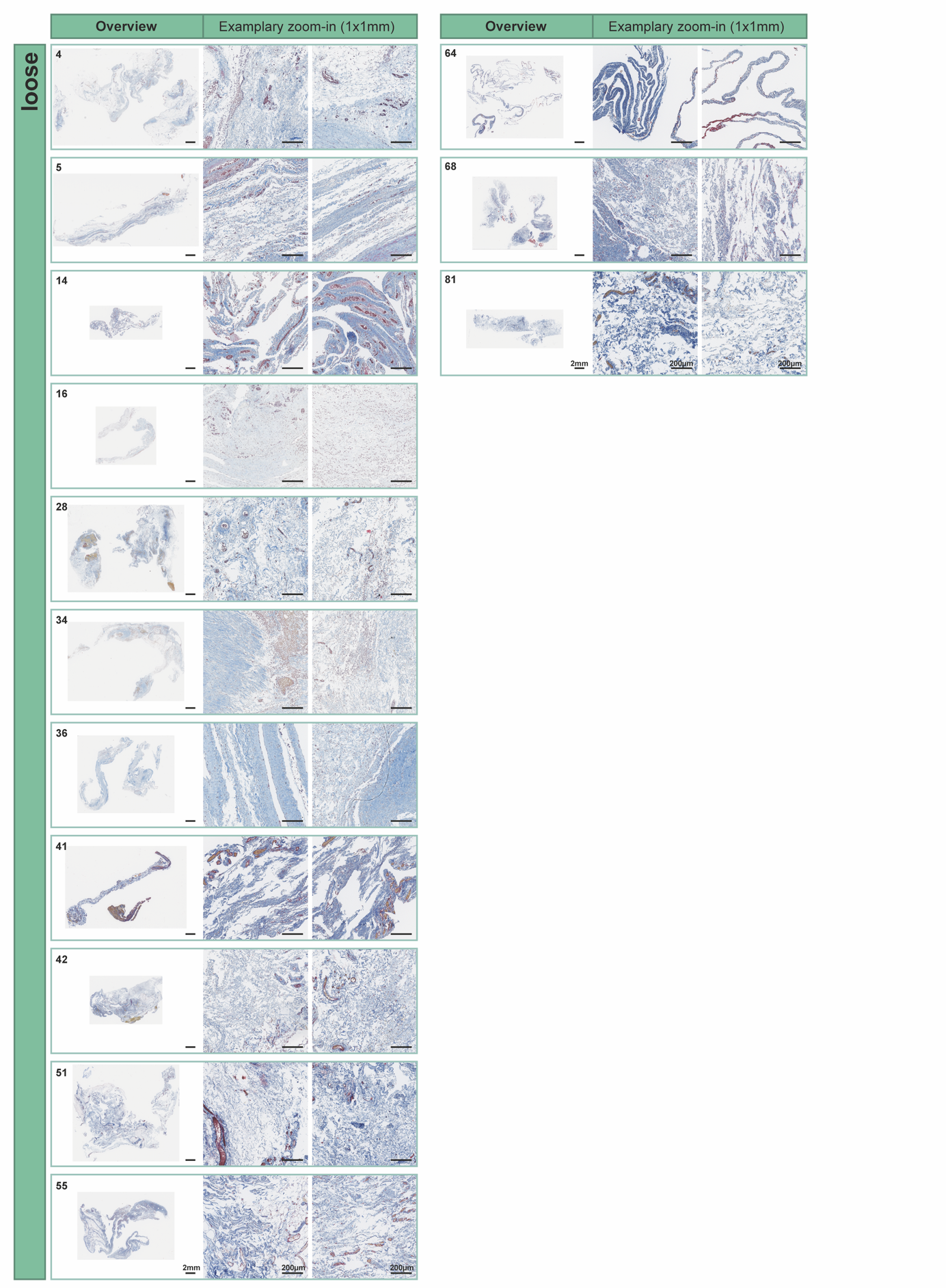


**Supplementary Figure 3:** Overview scans of all adhesion samples from the “loose” cluster with exemplary zoom-in sections (1x1mm) per sample. Scale bar = 2mm (overview) and 200µm (zoom-in).


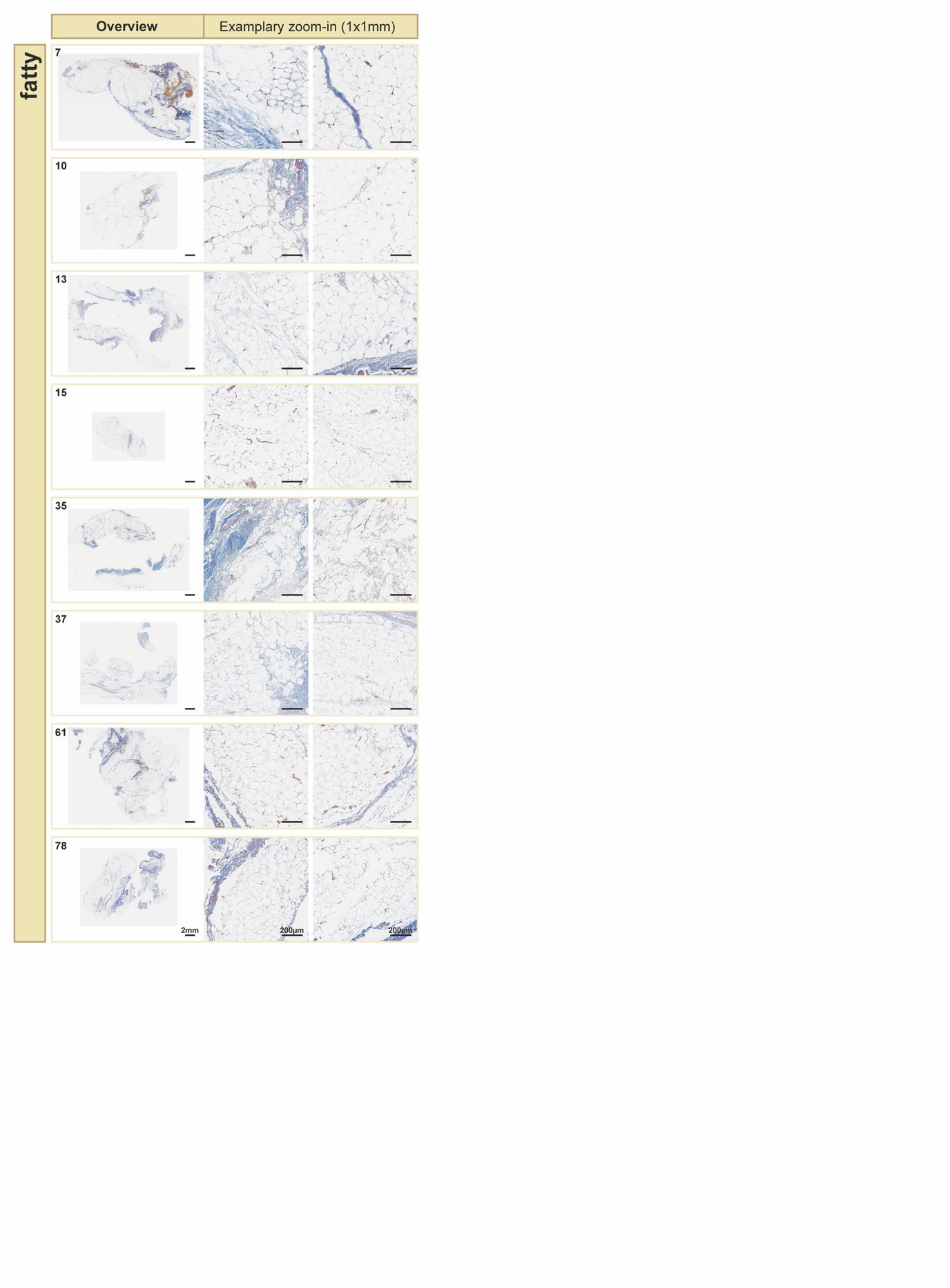


**Supplementary Figure 4:** Overview scans of all adhesion samples from the “fatty” cluster with exemplary zoom-in sections (1x1mm) per sample. Scale bar = 2mm (overview) and 200µm (zoom-in).


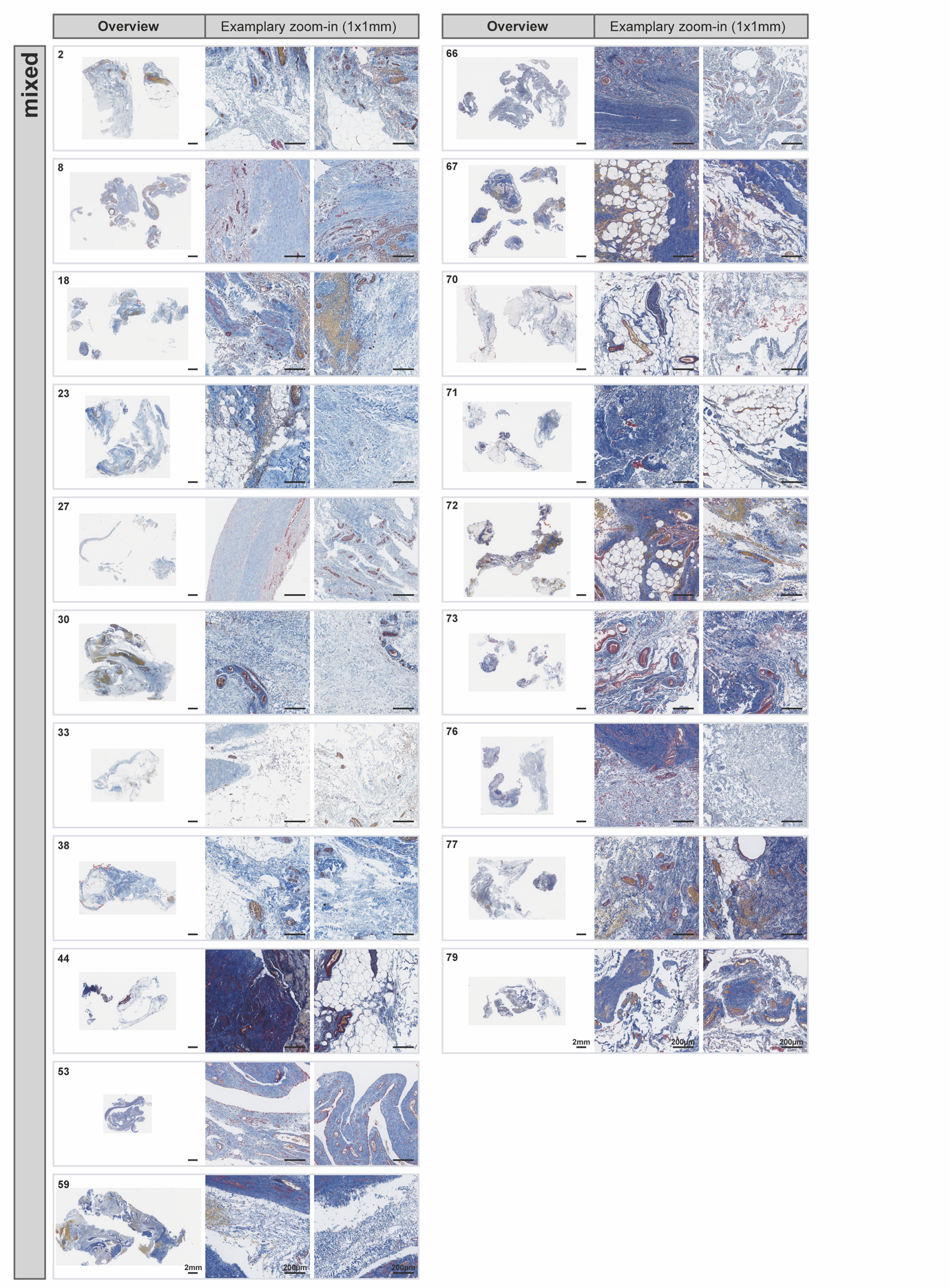


**Supplementary Figure 5:** Overview scans of all adhesion samples from the “mixed” cluster with exemplary zoom-in sections (1x1mm) per sample. Scale bar = 2mm (overview) and 200µm (zoom-in).
